# Synthetic Meta-Signal Observations: The Beidou Case

**DOI:** 10.3390/s24010087

**Published:** 2023-12-23

**Authors:** Daniele Borio, Ciro Gioia

**Affiliations:** 1 European Commission, Joint Research Centre (JRC), 21027 Ispra, Italy; 2 Independent Researcher, 21020 Brebbia, Italy; cirogioia@tin.it

**Keywords:** dual frequency, GNSS, meta-signals, measurement combinations

## Abstract

A Global Navigation Satellite System (GNSS) meta-signal is obtained when two or more side-band components from different frequencies are jointly processed as a single entity. This requires advanced signal processing techniques able to cope with the subcarrier, generated by the interaction of the side-band components, and handle possibly multi-peaked ambiguous correlation functions. An alternative approach to meta-signal processing is to reconstruct meta-signal observations using side-band measurements. Meta-signal high-accuracy pseudoranges can be reconstructed from the side-band code and carrier observations. The success of the reconstruction depends on several factors, including the frequency separation of the side-band components and the presence of measurement biases. The Beidou Navigation Satellite System (BDS), with its second- and third-generation signals, provides a wide range of components with various frequency separations. In this paper, we experimentally investigate the performance and limitations of the measurement reconstruction approach using Beidou observations. When the B1I and B1C components are considered, their reduced frequency separation leads to unambiguous measurements fully exploiting the potential of meta-signals. For larger frequency separations, jumps and discontinuities are observed in the position domain, which is a major limiting factor of this type of approach.

## 1. Introduction

A GNSS meta-signal is obtained when two or more components from different frequencies are processed and tracked together as a single entity [1]. In this way, a single signal with a large Gabor bandwidth is obtained. This is the pre-condition for obtaining high-accuracy pseudoranges and improved navigation performance [2]. While forming a GNSS meta-signal may lead to significant performance improvements, its effective processing requires advanced algorithms capable of fully exploiting the relationships between the different meta-signal components [3,4,5,6]. The large Gabor bandwidth of a GNSS meta-signal is a consequence of the frequency separation between the original side-band components used to form the meta-signal. While, in principle, a large frequency separation leads to a large Gabor bandwidth, the same frequency separation is the cause of multi-peaked correlation functions, which, in turn, can lead to ambiguous meta-signal pseudorange measurements. This is one of the main limitations of GNSS meta-signals [7,8,9]. The occurrence of a multi-peak correlation function and ambiguous measurements is directly linked to the fact that meta-signal pseudoranges are mixed code and carrier observations [10,11]. In particular, pseudoranges are effectively smoothed by subcarrier phase observations, which are a function of the side-band carrier phases.

Modern GNSSs broadcast signal components on several frequencies, representing a significant opportunity to form GNSS meta-signals and obtain improved Position Velocity and Timing (PVT) performance. Several research papers have developed advanced signal-processing methods [3,12] to process the Galileo Alternative Binary Offset Carrier (AltBOC) modulation [13], which combines the E5a and E5b side-band components into a single meta-signal. Also, the Galileo E6 signal can be combined with the E5 components to form meta-signals [6]. The BDS, with its wide range of second- and third-generation signals [14], provides several opportunities to jointly process components from different frequencies. The B1I/B1C signal combination is of particular interest for a small frequency separation of about 14 MHz. This combination has been analysed by several authors, who have proposed different signal-processing solutions [4,5,15,16], coherently combining the power received from the two side-band components. The benefits of wideband measurements from the B1I/B1C signal were recently demonstrated in [17] in the context of a single-frequency Precise Point Positioning (PPP) solution. The use of wideband B1I/B1C measurements significantly speeds up the PPP convergence time. The B1I/B1C case is not the only Beidou signal combination that can lead to effective performance improvements.

Although modern GNSSs provide several meta-signal opportunities, dedicated algorithms need to be implemented on the receiver side. This is required to fully exploit the potential of GNSS meta-signals. In the literature, two main categories of meta-signal receiver algorithms can be found. The first operates at the signal-processing and correlation level, whereas the second exploits and combines the side-band measurements. Signal-processing algorithms include the adoption of triple-loop receiver architectures [3,4,5,15,16], the use of the Least-squares AMBiguity Decorrelation Adjustment (LAMBDA) method [18], and collective unambiguous positioning approaches [12]. Triple-loop architectures are a direct extension of algorithms originally designed for the processing of Binary Offset Carrier (BOC) signals [19,20,21]. In this case, a dedicated loop is used to track the subcarrier component, and a bi-dimensional unambiguous correlation function is obtained. Although these approaches solve the ambiguity problem at the tracking level, the effective combination of pseudoranges and subcarrier phases needs to be implemented. In this respect, the LAMBDA method and collective unambiguous positioning use a triple-loop receiver architecture to obtain code and subcarrier measurements, which are then combined at the position solution level. The ambiguity problem is thus solved by the position engine, considering the spatial relationship between the different measurements, which are generated from the same receiver location. These approaches are usually computationally intensive and require significant receiver changes.

A second type of approach seeks to reconstruct GNSS meta-signal measurements from side-band observations [10,11]. This approach requires only limited receiver changes, and meta-signal measurements are computed from the side-band observations, which could be recorded in a standard Receiver INdependent EXchange (RINEX) format [22]. This also implies a reduction of the computational load for the processing of a full meta-signal. All these methods alleviate the ambiguity problem, which can still occur for excessively large frequency separations and in the presence of biases and non-idealities [7,8,9]. For this reason, it is important to investigate the limitations occurring when considering specific side-band combinations. In this respect, the meta-signal synthetic reconstruction approach was recently used to investigate the performance and limitations of Galileo meta-signals [23]. However, no analysis is currently available for BDS.

For this reason, this paper investigates the potential and limitations of the synthetic meta-signal measurement reconstruction approach applied to the Beidou system. In particular, an experimental analysis was conducted considering the different Beidou meta-signal combinations. Real measurements from a Septentrio PolaRx5 TR receiver (Leuven, Belgium) were collected from all Beidou frequencies and used in the analysis. Only the B2b signal was not analysed, as it is not supported by the Septentrio PolaRx5 TR receiver. The collected observations were processed using custom software to reconstruct the different meta-signal observations. The reconstructed measurements were then used to determine the user position using a Single Point Positioning (SPP) approach. Thus, meta-signal performance was assessed both in the measurement domain, with respect to the ambiguity resolution process, and in the position domain, in terms of horizontal and vertical errors. The data collection was conducted under static conditions.

From the experimental analysis, it emerged that meta-signal measurements can be effectively reconstructed for different BDS combinations. This is the case for the B1I/B1C and B2a/B3I combinations. The latter combination leads to very smooth high-accuracy pseudoranges with significantly improved position solutions. Also, the B2I/B3I combination shows promising results. Although the combinations involving the B2b signal were not analysed, they have significant potential, particularly when considered together with the B2a and B3I signals. Their analysis is left for future work.

The remainder of this paper is organised as follows. Section 2 summarises the meta-signal measurement reconstruction approach. Section 3 provides an overview of the different Beidou signals and dual-frequency combinations, whereas the experimental setup is described in Section 4. The experimental results are discussed in Section 5, and a discussion on possible extensions is provided in Section 6. Finally, some conclusions are drawn in Section 7.

## 2. Meta-Signal Measurement Reconstruction

The formulas for the reconstruction of the meta-signal observations were obtained from [10,23] by assuming a triple-loop architecture [3,5]. In this case, a single Delay Lock Loop (DLL) jointly tracks the codes of the two side-band components. A Phase Lock Loop (PLL) is used to process the mean carrier phase of the two side-band components, whereas a Subcarrier Phase Lock Loop (SPLL) is adopted to track their phase differences. As a consequence, three types of measurements are produced: pseudoranges from the DLL and carrier and subcarrier phases from the PLL and SPLL, respectively. The reconstruction formulas, which are briefly summarised below, are obtained by comparing the outputs of a triple-loop architecture with the measurements obtained from a standard receiver configuration, where side-band signals are processed independently.

### 2.1. Carrier Phase

According to [10,23], the meta-signal carrier phase tracked by the triple-loop PLL can be reconstructed as the average side-band carrier phase:

(1) $$\varphi_{0}=\frac{1}{2}\left(\varphi_{1}+\varphi_{2}\right)$$

where $\varphi_{0}$ is the meta-signal carrier phase. $\varphi_{1}$ and $\varphi_{2}$ are the side-band carrier phases, and all the measurements in (1) are expressed in cycles. Equation (1) can be expressed in metres as:

(2) $$\begin{array}{cc} \Phi_{0} & =\frac{f_{1}\Phi_{1}+f_{2}\Phi_{2}}{f_{1}+f_{2}}, \end{array}$$

where $\Phi_{0}$, $\Phi_{1}$, and $\Phi_{2}$ are the carrier phase measurements expressed in metres. They are obtained by multiplying $\varphi_{0}$, $\varphi_{1}$, and $\varphi_{2}$ by the respective wavelengths: $\lambda_{0}$ for the meta-signal and $\lambda_{1}$ and $\lambda_{2}$ for the side-bands. In (2),

(3) $$f_{1}=\frac{c}{\lambda_{1}},\;f_{2}=\frac{c}{\lambda_{2}}$$

are the side-band centre frequencies. *c* is the speed of light, and $\lambda_{0}$ is obtained as $\frac{c}{f_{0}}$, with $f_{0}$ being the average of the two side-band centre frequencies. The meta-signal carrier phase is the narrow-lane carrier phase combination [24] of the side-band carrier phase observations.

### 2.2. Subcarrier Phase

Subcarrier phase observations are obtained as the average side-band phase difference [10,23]:

(4) $$\varphi_{sub}=\frac{1}{2}\left(\varphi_{2}-\varphi_{1}\right)$$

where $\varphi_{sub}$ is the subcarrier phase in cycles. When expressed in meters, the subcarrier phase becomes:

(5) $$\Phi_{sub}=\frac{f_{2}\Phi_{2}-f_{1}\Phi_{1}}{f_{2}-f_{1}}$$

where $\Phi_{sub}=\varphi_{sub}\lambda_{sub}$, and $\lambda_{sub}$ is the subcarrier wavelength defined as

(6) $$\lambda_{sub}=\frac{c}{f_{sub}}.$$

The subcarrier frequency is computed as

(7) $$f_{sub}=\frac{f_{2}-f_{1}}{2}.$$

Equation (4) shows that $\varphi_{sub}$ is the wide-lane combination [24] of the side-band carrier phase observations.

The relationships provided above are derived from the definitions of the subcarrier component. However, it is important to note that the division by 2 in (4) leads to measurements with half-cycle ambiguities. This condition corresponds to triple-loop architectures adopting Costas-like SPLL discriminators [3]. In this case, the SPLL is not able to solve full subcarrier cycles. If an ambiguity resolution process is attempted, as discussed further in the following section, half-wavelength should be considered. Thus, one should consider the wavelength of the wide-lane carrier combination:

(8) $$\lambda_{wl}=\frac{\lambda_{sub}}{2}.$$

### 2.3. Pseudorange Measurements

For meta-signals, two types of pseudoranges can be computed: raw and high-accuracy pseudoranges. The former are code-only measurements and are reconstructed as:

(9) $$\rho_{0}=\frac{\alpha_{1}\sqrt{\frac{C_{1}}{N_{0}}}}{\alpha_{1}\sqrt{\frac{C_{1}}{N_{0}}}+\alpha_{2}\sqrt{\frac{C_{2}}{N_{0}}}}\rho_{1}+\frac{\alpha_{2}\sqrt{\frac{C_{2}}{N_{0}}}}{\alpha_{1}\sqrt{\frac{C_{1}}{N_{0}}}+\alpha_{2}\sqrt{\frac{C_{2}}{N_{0}}}}\rho_{2},$$

where $\rho_{0}$ is the reconstructed raw pseudorange, and $\rho_{1}$ and $\rho_{2}$ are the pseudoranges obtained for the side-band components. $\frac{C_{1}}{N_{0}}$ and $\frac{C_{2}}{N_{0}}$ are the Carrier-To-Noise Power Spectral Density Ratios ($C/N_{0}$s) of the side-band components, and $\alpha_{1}$ and $\alpha_{2}$ are the slopes of the main correlation peaks of the side-band components [23]. $C/N_{0}$ values have to be expressed in linear units.

For symmetric modulations, such as the AltBOC, for side-band components received with the same $C/N_{0}$, (9) becomes the mean pseudorange:

(10) $$\rho_{0}=\frac{1}{2}\left(\rho_{1}+\rho_{2}\right).$$

High-accuracy pseudoranges are reconstructed as in [23]:

(11) $$\rho^{+}=\Phi_{sub}+round\left(\frac{\rho_{0}-\Phi_{sub}}{\lambda_{wl}}\right)\lambda_{wl}.$$

where $\rho^{+}$ is the high-accuracy pseudorange in metres.

The rounding operation in (11) corresponds to an ambiguity resolution process and takes into account the fact that synthetic observations are affected by half-cycle ambiguities. For this reason, $\lambda_{wl}$ is used instead of $\lambda_{sub}$.

### 2.4. Measurement Pre-Correction

Several error sources [24,25] can introduce biases and affect the quality of the final reconstructed measurements (11). A possibility for reducing these biases is to apply corrections to the side-band measurements. Similarly to [23], we considered the application of such corrections to the measurements before computing the different meta-signal combinations. We denoted this process as “measurement pre-correction”.

The following corrections were applied:

- Ionospheric corrections based on the Klobuchar model [26];
- Tropospheric corrections based on the Saastamoinen model [27];
- Satellite clock corrections based on the polynomial model, whose parameters are broadcast as part of the Beidou navigation message [28,29];
- Relativistic and Sagnac effects [25];
- Group delay corrections based on parameters broadcast in the Beidou navigation message [28,29].

All the corrections listed above also have to be applied to the solutions using reconstructed measurements. In the case of measurement pre-correction, these corrections are applied directly to the side-band components. Thus, corrections affect both $\rho_{0}$ and $\Phi_{sub}$ in (11) and enter the ambiguity resolution process implemented through rounding. In the standard case, corrections are directly applied to $\rho^{+}$ and do not affect the ambiguity resolution process. Note that the same models are applied to pseudoranges and carrier phases. Thus, the main difference between the two cases is with respect to the argument of the rounding operation in (11).

It is also important to note that the normalised Undifferenced (UD) code-carrier combinations,

(12) $$CCC=\frac{\rho_{0}-\Phi_{sub}}{\lambda_{wl}},$$

are strictly related to the Hatch Melbourne Wübbena (HMW) combinations [23,30]. HMW combinations are iono-free and thus insensitive to ionospheric corrections that cancel out when forming the combination. This is also approximately true for (12). For ionospheric errors, the corrections were scaled with respect to a factor dependent on the square of the centre frequencies of the different components. For small frequency separations, the scaling factor converges to one, and the ionospheric effect on the two side-band components can be considered the same.

While a limited impact of pre-corrections is expected, this fact is empirically verified in the following section.

## 3. Beidou Signal Overview

**Figure 1:**
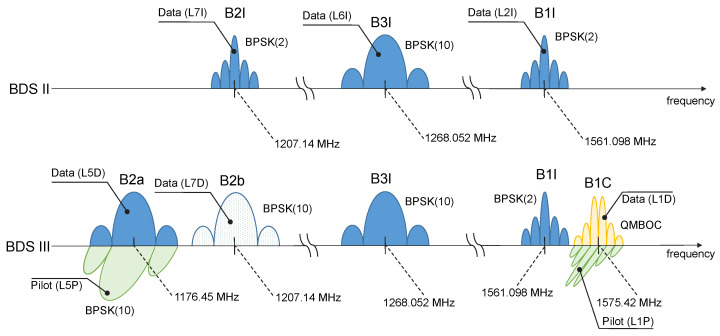
Schematic representation of the Beidou open signal frequency plan. Both BDS-2 and BDS-3 signals are represented.

The BDS constellation is currently made by a combination of second- (BDS-2) and third (BDS-3)-generation satellites, each broadcasting signals on different frequencies [14,31]. A schematic representation of the different BDS-2 and BDS-3 signals is provided in Figure 1. Second-generation satellites broadcast open signals in three frequency bands with different characteristics. All BDS-2 signals are data components, whereas BDS-3 introduces new advanced modulations, including pilot channels [14]. Figure 1 also provides the signal centre frequencies, the signal codes adopted by the Beidou Interface Control Document (ICD) [28,29], and the codes used in the RINEX format to identify carrier phase observations [22]. The B3I and B1I signals are maintained when passing from the second to the third generation and thus are broadcast from all Beidou satellites. On the contrary, the B2I signal, in the band corresponding to the Galileo E5b frequency, has been replaced with a new component, the B2b one, which is broadcast together with the B2a signal to form the Asymmetric Constant-Envelope Binary Offset Carrier (ACE-BOC) modulation [32]. For this reason, the B2I signal is broadcast only by second-generation satellites.

The B2a, B2b, and B1C components are available only from third-generation satellites. The Quadrature Multiplexed Binary Offset Carrier (QMBOC) modulation [14] is broadcast in the B1C frequency band and features a data (L1D RINEX code) and a pilot (L1P RINEX code) component. Given this complex signal landscape and the fact that current receivers may not be able to process all Beidou modulations, the possibility of forming dual-frequency combinations is limited by the measurement availability. This fact is further discussed in Section 4, where the setup adopted for the experimental analysis is described. More specifically, the receiver used for data collection does not support the B2b signal, and only B2I components are processed.

Figure 1 also indicates whether signals are broadcast in-phase or in quadrature. New BDS-3 pilot signals are transmitted with a $\frac{\pi }{2}$ phase offset, which corresponds to a quarter of cycle phase difference with respect to the data components. This offset has to be corrected when reconstructing meta-signal observations. The reference code and phase alignment for the different Beidou signals can be found in Table A23 in [22].

**Figure 2:**
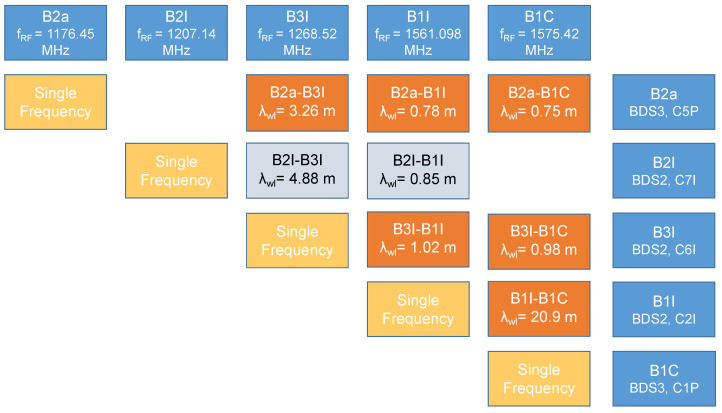
Different Beidou meta-signal combinations considered in this paper, along with the associated wide-lane wavelengths.

The different Beidou meta-signal combinations considered in this paper are briefly summarised in Figure 2, which also provides the resulting wide-lane wavelengths used for the reconstruction of high-accuracy pseudoranges.

In Figure 2, it is evident that a wide range of wavelengths, from about 20 m to less than 1 m, are available for the Beidou case. The BI1/B1C combination is of particular interest since it is characterised by the largest wavelength of more than 20 m. This wavelength allows for a more effective ambiguity resolution process, which is further investigated in Section 4. In Figure 2, the combinations involving the B2I signal and the B2a and B1C components are missing. This is due to the fact that the B2I signal is second generation and is not broadcast by BDS-3 satellites. Since both B2a and B1C components are third-generation signals, it is not possible to form combinations with the B2I component. The B2I/B3I and B2I/B1I combinations are possible and available when considering signals broadcast by BDS-2 satellites. In Figure 2, these combinations are depicted in grey.

## 4. Experimental Setup

**Figure 3:**
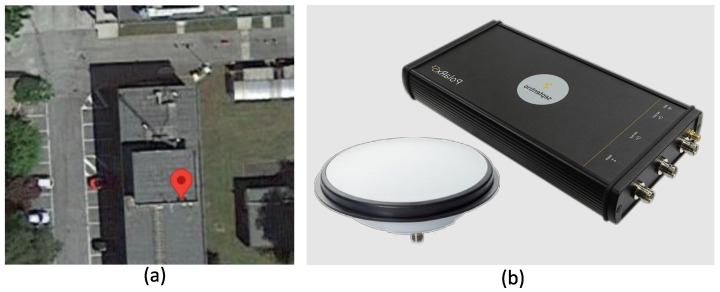
Experimental setup adopted for the analysis of synthetic Beidou meta-signal observations. (**a**) View of the location of the static antenna used for data collection. (**b**) Receiver and antenna used for data collection.

In order to analyse the Beidou meta-signal combinations depicted in Figure 2, the same experimental setup adopted in [23] for the study of the Galileo case was considered. The setup is shown in Figure 3. In particular, a Septentrio PolaRx5 TR receiver was adopted along with a Novatel GNSS-850 multi-band professional antenna (Calgary, Canada), which was placed on the rooftop of the building shown in Figure 3a. A view of the Septentrio PolaRx5 TR receiver and the Novatel GNSS-850 antenna is provided in Figure 3b.

The experiment involved the collection of four hours of data with a 1 Hz rate. Measurements from all Beidou frequencies were collected. Only the B2b signal was not recorded since it was not supported by the Septentrio PolaRx5 TR receiver. Given the lack of B2b measurements, it was not possible to analyse the combination related to the ACE-BOC, which is the Beidou equivalent of the Galileo AltBOC.

**Figure 4:**
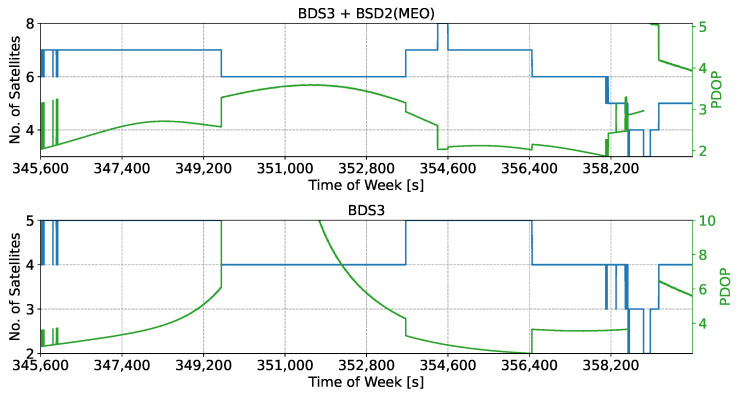
Number of satellites (blue lines) used in the PVT solution and the associated PDOP (green lines). (**Upper box**) Both BDS-2 MEO and BDS-3 satellites are considered, leading to higher signal availability. (**Lower box**) BDS-3 case: results obtained considering only the signals broadcast by BDS-3 satellites.

While the signals were collected under a clear-sky environment, different geometric conditions were obtained depending on the signal combination considered. The availability conditions are analysed in Figure 4, which shows the number of satellites used in the PVT and the corresponding Position Dilution of Precision (PDOP). Two cases are analysed in Figure 4; the best visibility/availability scenario is considered in the upper box of the figure. It was obtained considering signals from both the BDS-2 Medium Earth Orbit (MEO) and BDS-3 satellites. This is, for instance, the case for the B3I/B1I combination, as the two underlying signals were broadcast from both types of satellites, leading to a higher availability. In this case, up to eight satellites were available for computing the final position solution. The corresponding PDOP was practically always lower than 5.

Note that in all cases, satellites with elevations lower than 10 degrees were excluded from the navigation solution.

The bottom part of Figure 4 analyses the case where only signals from BDS-3 satellites were considered. This is, for instance, the case for the B2a/B1C combination, as the underlying signals are not available in second-generation satellites. The use of only BDS-3 satellites reduced the signal availability and worsened the geometric conditions of the experiment. In particular, a PDOP greater than 5 was observed for a significant portion of the test. Epochs characterised by PDOP values greater than 5 were not considered in the analysis discussed below.

The different Beidou meta-signal combinations and the associated satellite visibility conditions are summarised in Table 1. Combinations associated with the “BDS-2 and 3” label in Table 1 are characterised by the geometric conditions described in the upper part of Figure 4, whereas the label “BDS-3” refers to the bottom box of the figure. The combinations involving the B2I signals were obtained using only second-generation satellites. The geometric conditions were determined considering only MEO satellites. For the Geosynchronous Equatorial Orbit (GEO) and Inclined Geosynchronous Orbit (IGSO) satellites, results are provided in terms of the ambiguity resolution capabilities.

## 5. Experimental Results

In this section, the experimental results obtained using the synthetic meta-signal approach are presented for the different Beidou cases discussed above. The B1I/B1C case, which is the most attractive for the small frequency separation of its side-band components, is analysed first. Other combinations are then analysed.

The analysis is performed in terms of the quality of the reconstructed measurements and performance in the position domain. In particular, the final quality of high-accuracy pseudoranges (11) depends on the bias and standard deviation of normalised UD code-carrier combinations (12). These combinations are experimentally analysed in the following subsection.

The location of the antenna used for the data collection was accurately surveyed. In this way, it was possible to determine the actual position errors obtained considering an SPP solution based on the reconstructed measurements. These errors are also analysed in the following subsection.

### 5.1. The B1I/B1C Combination

**Figure 5:**
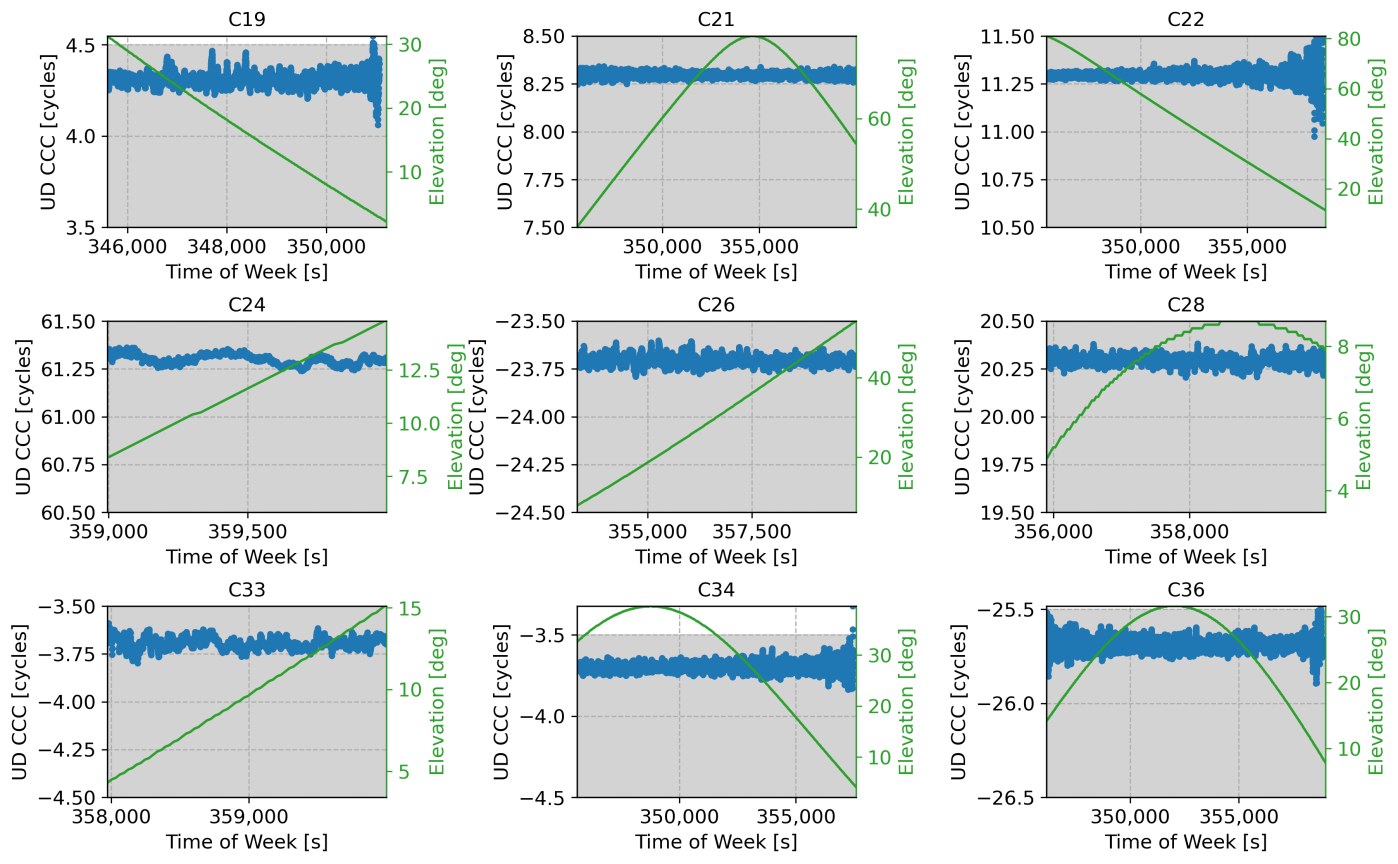
UD code-carrier combinations used for the ambiguity resolution process for the different satellites broadcasting both B1I and B1C signals, expressed in cycle counts. The corresponding satellite elevation is also provided in green.

The UD code-carrier combinations obtained for the B1I/B1C case are depicted in Figure 5.

The combinations were obtained by applying a quarter of cycle correction, as specified in [22]. Despite this, a clear common Fractional Bias (FB) is evident in Figure 5. The FB is defined as

(13) FB=CCC−round(CCC)

and should be ideally close to zero to allow for an effective ambiguity resolution through rounding. Indeed, the FB quantifies the distance of a code-carrier combination to its closest integer. In this case, a common FB of about 0.3 cycles affected all code-carrier combinations. This is better highlighted in Table 2, which provides summary statistics for the different code-carrier combinations.

In this case, the FB did not affect the rounding operations since it was still sufficiently far away from the critical boundary values at around ±0.5. Moreover, the B1I/B1C code-carrier combinations were affected by relatively low standard deviations in the order of 0.028 cycles. Thus, the noise was reduced with respect to the B1I/B1C wide-lane wavelength and did not cause ambiguities in the rounding operations. A summary of the standard deviations observed for the different satellites is also provided in Table 2.

**Figure 6:**
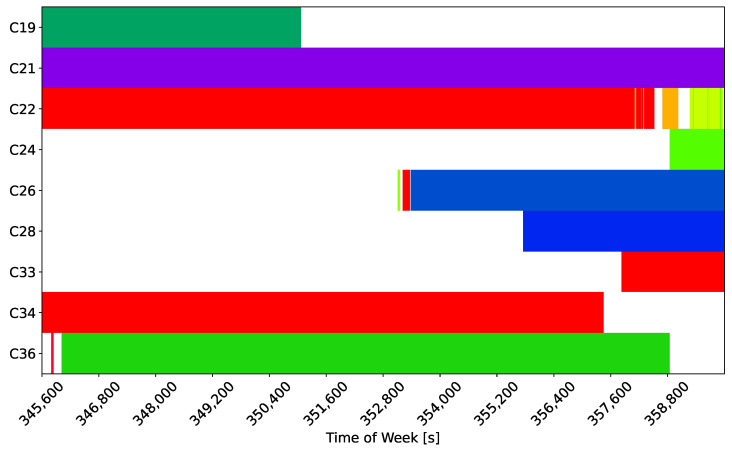
Graphical representation of the integer number of cycles estimated using the rounding operation in (11) for the different satellites: B1I/B1C case.

The effect of rounding on the UD code-carrier combinations is studied in Figure 6 for the B1I/B1C case. The figure provides a graphical representation of the integer number of cycles estimated using the rounding operation in (11) for the different satellites. The colour scheme has been selected in order to highlight possible cycle slips due to the noise and bias on the code-carrier combinations.

From the figure, it is clear that all the signals were locked on a practically constant integer value and that variations occurred only for low-elevation satellites when the receiver was acquiring or losing signal lock. This result confirms that the large $\lambda_{wl}$ of the BI1/B1C combination allows one to effectively and steadily solve the ambiguities in the high-accuracy pseudorange reconstruction process. While the identification of the root causes of the FB observed for the code-carrier combinations in Figure 5 needs further investigation, it does not compromise the outcome of the rounding operations in the BI1/B1C case, as evident in Figure 6.

The application of pre-corrections to the code-carrier combinations only marginally affected them. In particular, no changes were observed with respect to the rounding process. This was expected since the B1I and B1C are very close in frequency and affected by similar corrections, which were cancelled out when applying the reconstruction formula (11). This confirms the expectations introduced in Section 2.4.

**Figure 7:**
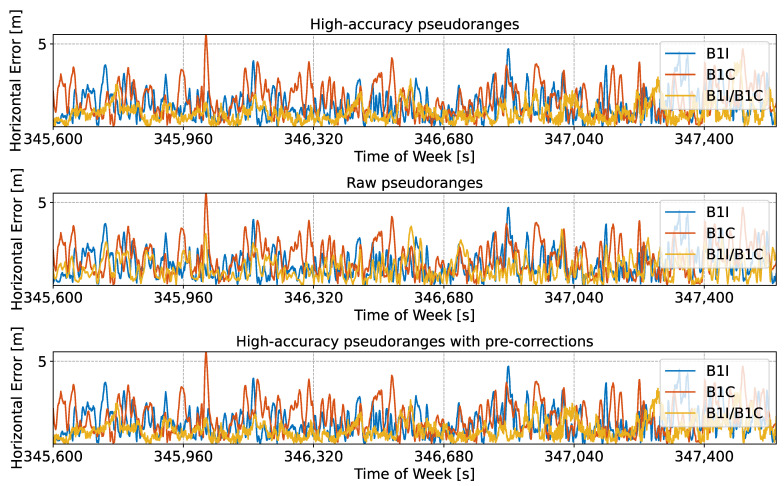
Time evolution of the horizontal error obtained using an SPP solution for different meta-signal measurements. The single side-band solutions are also provided for comparison purposes. B1I/B1C combination. (**Upper box**) Solution obtained using high-accuracy pseudoranges. (**Middle box**) Solution obtained using raw pseudoranges. (**Lower Part**) Solution obtained using high-accuracy pseudoranges computed by applying pre-corrections.

**Figure 8:**
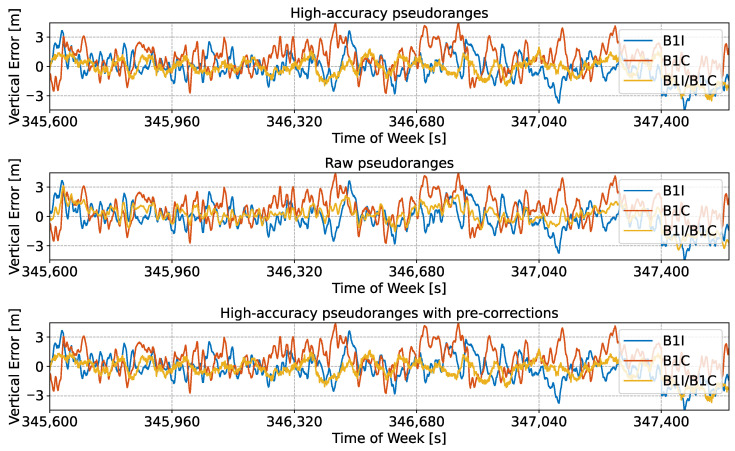
Time evolution of the vertical error obtained using an SPP solution for different meta-signal measurements. The single side-band solutions are also provided for comparison purposes. B1I/B1C combination. (**Upper box**) Solution obtained using high-accuracy pseudoranges. (**Middle box**) Solution obtained using raw pseudoranges. (**Lower box**) Solution obtained using high-accuracy pseudoranges, computed by applying pre-corrections.

The high-accuracy pseudoranges reconstructed from the code-carrier combinations analysed in the previous figures were used to compute an SPP solution based on a Weighted Least-Squares (WLS) approach. The obtained position solutions were compared to the actual antenna location, leading to the errors shown in Figure 7 and Figure 8.

Figure 7 shows the time evolution of the horizontal error, along with the errors obtained considering the original side-band observations.

Three cases are analysed in the figure. The solution obtained using high-accuracy pseudoranges is considered in the upper part of the plot, whereas the solution obtained using raw pseudoranges is provided in the middle box of the figure. Finally, high-accuracy pseudoranges computed by applying pre-corrections are depicted in the lower box of the figure. The first 30 min of the test were considered to improve the clarity of the plot. In all three cases, the reconstructed meta-signal observations provided improved performance with respect to single side-band processing.

Similar conclusions can be drawn when considering the vertical error, which is studied in Figure 8. In this case, three cases were also analysed: high-accuracy pseudoranges, raw pseudoranges, and measurements with pre-corrections. While the greatest benefits were obtained when using high-accuracy pseudoranges, raw observations provided some benefits compared to the side-band solutions. Also, the results in the position domain confirm that pre-corrections have a limited impact on the reconstruction process and the final position performance.

It should be noted that in both Figure 7 and Figure 8, no jumps can be observed. Jumps and solution discontinuities were observed in [23] for some of the Galileo meta-signal combinations due to difficulties in the ambiguity resolution process. These phenomena, which occurred for other Beidou meta-signal combinations, did not occur in the B1I/B1C case.

Summary statistics for the horizontal and vertical errors analysed in Figure 7 and Figure 8 are provided in Table 3.

The table includes the mean absolute error, Root-Mean-Square (RMS) error, and standard deviation. It is important to note that these statistics include all error sources, including orbital and clock errors, which cannot be mitigated by the meta-signal approach. The meta-signal combinations reduced multipath and noise-related errors. Despite this, the benefits of the meta-signal approaches are evident in Table 3. For the horizontal case, the mean absolute error was reduced by more than half a meter when passing from the B1C solution to the one computed using raw pseudoranges. The error was almost halved when using high-accuracy pseudoranges. As already discussed, only marginal differences between the cases with and without pre-corrections were observed. The standard deviations obtained using meta-signal combinations were also reduced by about 30% with respect to the single-frequency solutions. Similar conclusions can be drawn for the vertical channel: for all cases based on meta-signals, sub-meter mean absolute error values were found. This accuracy was not achieved by the single side-band solutions. These statistics confirm the fact that the reconstruction approach provides a simple mechanism to access wideband measurements from the B1 frequency without requiring complex signal processing algorithms, as in [17].

**Figure 9:**
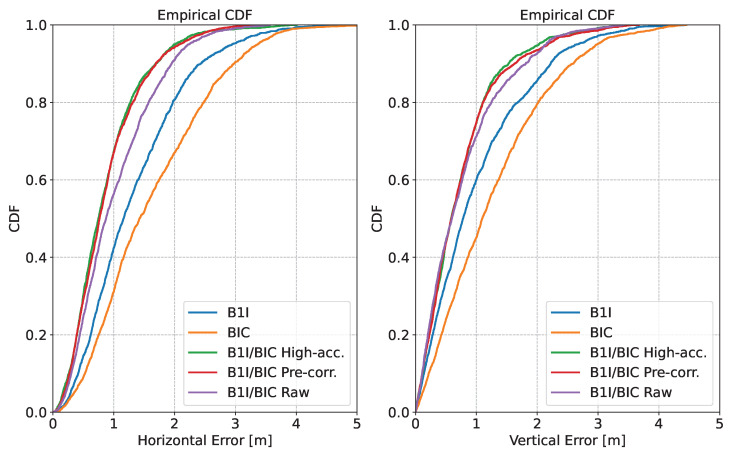
CDFs of the different combinations obtained using the B1I and B1C signals. (**Left box**) Horizontal error. (**Right box**) Absolute value of the vertical error.

The performance obtained in the position domain is further analysed in Figure 9, which shows the Cumulative Density Functions (CDFs) of the different combinations obtained using the B1I and B1C signals. The left box of the figure considers the horizontal error, whereas the vertical channel is analysed in the right part.

The benefits of reconstructed meta-signal measurements are evident in the figure. Both horizontal and vertical errors were reduced with respect to single side-band processing. The 95-percentile horizontal error was reduced by about one meter when moving from the B1I single-frequency solution to the one obtained using high-accuracy pseudoranges. As already mentioned, the impact of the pre-corrections was limited. The use of raw pseudoranges was also beneficial, even though the full potential of the meta-signals was not exploited.

**Figure 10:**
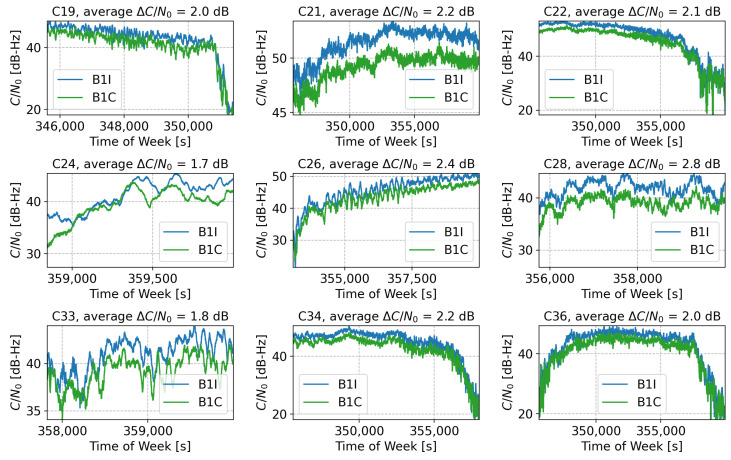
Comparison between the B1I and B1C $C/N_{0}$ values recorded for the different satellites visible during the experiment. The figure also reports the average $C/N_{0}$ differences, denoted as $\Delta C/N_{0}$.

From the results reported above, it is evident that the B1C single-frequency solution led to worse positioning performance compared to the corresponding B1I solution. While the B1C signal has been designed to have a larger Gabor bandwidth compared to the B1I component [15], it is received with lower power [14]. This is confirmed by the results reported in Figure 10, which compares the B1I and B1C $C/N_{0}$ values recorded for the different satellites visible during the experiment. The figure also provides the average $C/N_{0}$ differences, denoted as $\Delta C/N_{0}$.

From the comparison, it is evident that the B1I signals were received with a $C/N_{0}$ gain of about 2 dBs with respect to the corresponding B1C component. These results are in agreement with the findings reported in [14]. The $C/N_{0}$ gain of the B1I signals justifies the improved performance observed in the position domain with respect to the B1C component.

### 5.2. The B2a/B3I Combination

**Figure 11:**
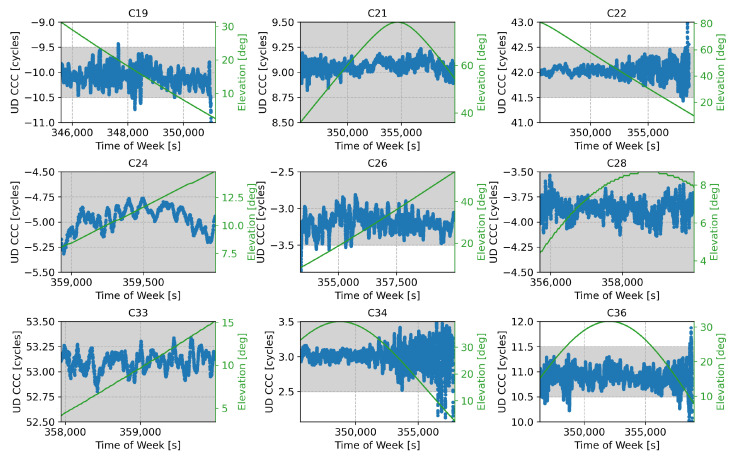
UD code-carrier combinations used for the ambiguity resolution process for the different satellites broadcasting both B2a and B3I signals, expressed in cycle counts. The corresponding satellite elevation is also provided in green.

The B2a/B3I combination was characterised by a frequency separation of 92.07 MHz with a wide-lane wavelength of about 3.26 m. The UD code-carrier combinations for the B2a/B3I case are provided in Figure 11, expressed in cycle counts, along with the elevations of the corresponding satellites.

In this case, no common FB was observed. The code-carrier combinations were approximately aligned around the integer values and could be effectively used for the ambiguity resolution process. This is confirmed by the summary statistics provided in Table 4. Only satellites with Pseudo-Random Number (PRN)s of 26, 28, and 33 showed a slightly increased bias. However, the biases observed were below 0.2 cycles and did not impact the ambiguity resolution process implemented through rounding. Satellites 28 and 33 are low elevation and should not be used in the computation of the final navigation solution. The standard deviations of the different combinations increased with respect to the B1I/B1C case, reaching values up to about 0.2 cycles. This value was nearly one order of magnitude greater than the standard deviations observed for the B1I/B1C case.

Despite the increased standard deviations, the ambiguities could still be solved in a reliable way. This can be clearly seen in Figure 11, where the code-carrier combinations are within the grey bands, representing the region that will be mapped to a single integer value through the rounding operation. The B2a/B3I code-carrier combinations in Figure 11 only sporadically exist within the grey bands. Moreover, this happens mostly for low-elevation values, where the noise affecting the combinations is stronger.

**Figure 12:**
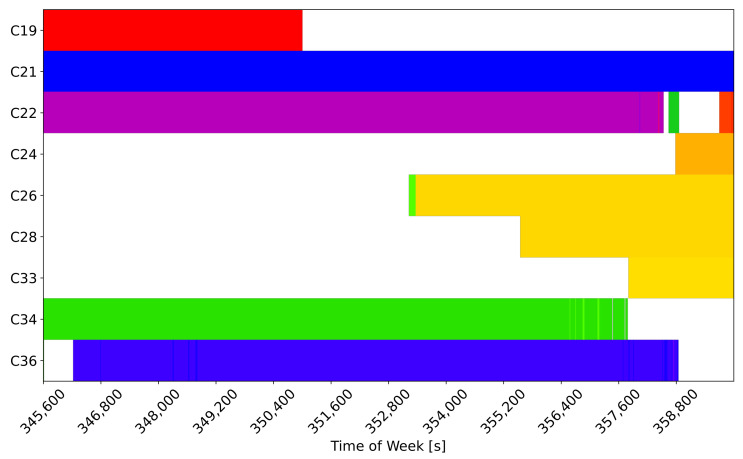
Graphical representation of the integer number of cycles estimated using the rounding operation in (11) for the different satellites. B2a/B3I case.

The fact that ambiguities could be reliably solved in the B2a/B3I case is also evident in Figure 12, which provides a graphical representation of the integer number of cycles estimated for the different code-carrier combinations analysed in Figure 11. For the B1I/B1C case, ambiguities were practically always resolved to the same value, and only sporadic jumps were observed. These jumps mainly occurred when the satellite entered or exited the visibility period at low-elevation angles or when the receiver encountered difficulties maintaining stable carrier phase tracking for low $C/N_{0}$ values.

These results confirm that B2a and B3I measurements can be effectively combined to obtain high-accuracy pseudoranges. The relatively small frequency separation and the lack of FB allowed for a reliable ambiguity resolution process and an effective measurement reconstruction.

**Figure 13:**
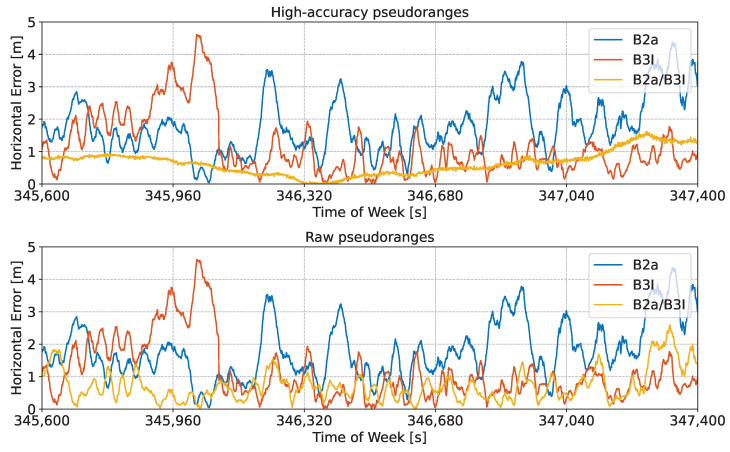
Time evolution of the horizontal error obtained using an SPP solution for different meta-signal measurements. The single side-band solutions are also provided for comparison purposes. B2a/B3I combination. (**Upper box**) Solution obtained using high-accuracy pseudoranges. (**Lower box**) Solution obtained using raw pseudoranges.

In turn, the reconstructed measurements allowed for the computation of accurate position solutions. This is evident in Figure 13, which shows the time evolution of the horizontal error obtained using an SPP solution for the different meta-signal measurements determined for the B2a/B3I case. The use of pre-corrections was not further analysed since the differences with respect to the standard case (with no pre-corrections) were limited.

High-accuracy pseudoranges led to a very smooth horizontal solution, with errors varying gradually with time. Since a standard SPP solution was implemented, the residual orbital, clock, and atmospheric errors were not compensated for. These elements justify the residual bias present in the horizontal error analysed in the bottom part of Figure 13. Despite this, the benefits of the meta-signal approach for the B2a/B3I combination are evident, as the horizontal error was significantly reduced and always below two meters.

The bottom part of Figure 13 compares the horizontal error obtained with raw pseudoranges to the single side-band solutions. Although in this case, clear performance improvements were also observed, the full potential of the meta-signal approach was not exploited. This is due to the fact that the benefits of the subcarrier were not exploited.

**Figure 14:**
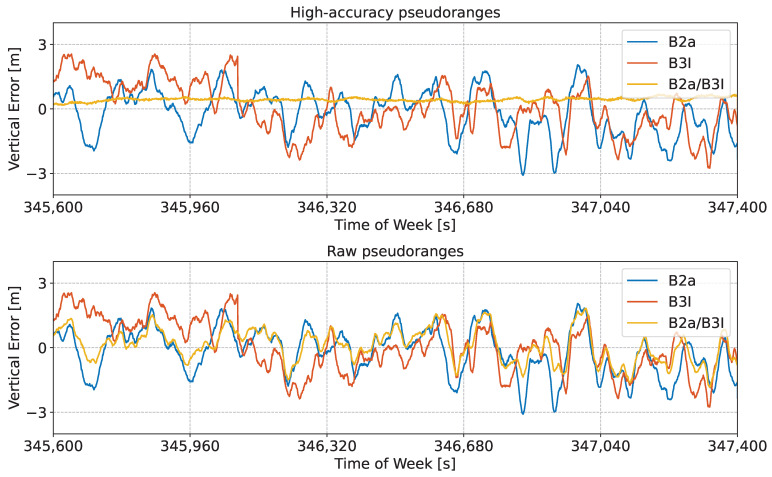
Time evolution of the vertical error obtained using an SPP solution for different meta-signal measurements. The single side-band solutions are also provided for comparison purposes. B2a/B3I combination. (**Upper box**) Solution obtained using high-accuracy pseudoranges. (**Lower box**) Solution obtained using raw pseudoranges.

Similar conclusions can be drawn from Figure 14, which shows the time evolution of the vertical error obtained using an SPP solution for the B2a/B3I meta-signal combinations.

In this case, the performance improvements due to the use of high-accuracy pseudoranges are also evident. The vertical error depicted in the upper box of Figure 14 is very stable with reduced oscillations. It is always below 50 cm, with a standard deviation of about 11 cm. The single side-band solutions reached vertical errors of more than 3 m, with standard deviations of around 60 cm. The vertical error obtained when considering the raw pseudoranges is analysed in the lower part of Figure 14. For the horizontal error, the raw pseudoranges provided some improvements with respect to the single side-band cases. However, this improvement was significantly reduced with respect to the high-accuracy case. When ambiguities could be reliably solved, the subcarrier provided a significant positive contribution toward the computation of high-accuracy measurements.

These results are confirmed by the summary statistics reported in Table 5 for the B1a/B3I combination. The wide-lane wavelength of the B2a/B3I combination is a good compromise in terms of ambiguity resolution and measurement smoothing. Indeed, the B2a/B3I wide-lane wavelength was almost seven times smaller compared to that of the B1I/B1C case. A smaller wavelength provided better smoothing of the raw pseudoranges at the expense of the ambiguity resolution process, which could still be successfully implemented. The lack of significant FB was also a positive element supporting the reconstruction approach.

### 5.3. The B2I/B3I Combination

The B2I/B3I combination was obtained considering signals transmitted by second-generation satellites. In BDS-3, the B2I signal was replaced with the B2b component [14]. In this case, the frequency separation was 61.38 MHz with a wide-lane wavelength of about 4.88 m. This wavelength is larger than that of the B2a/B3I combination and thus it has the potential to enable reliable ambiguity resolution.

**Figure 15:**
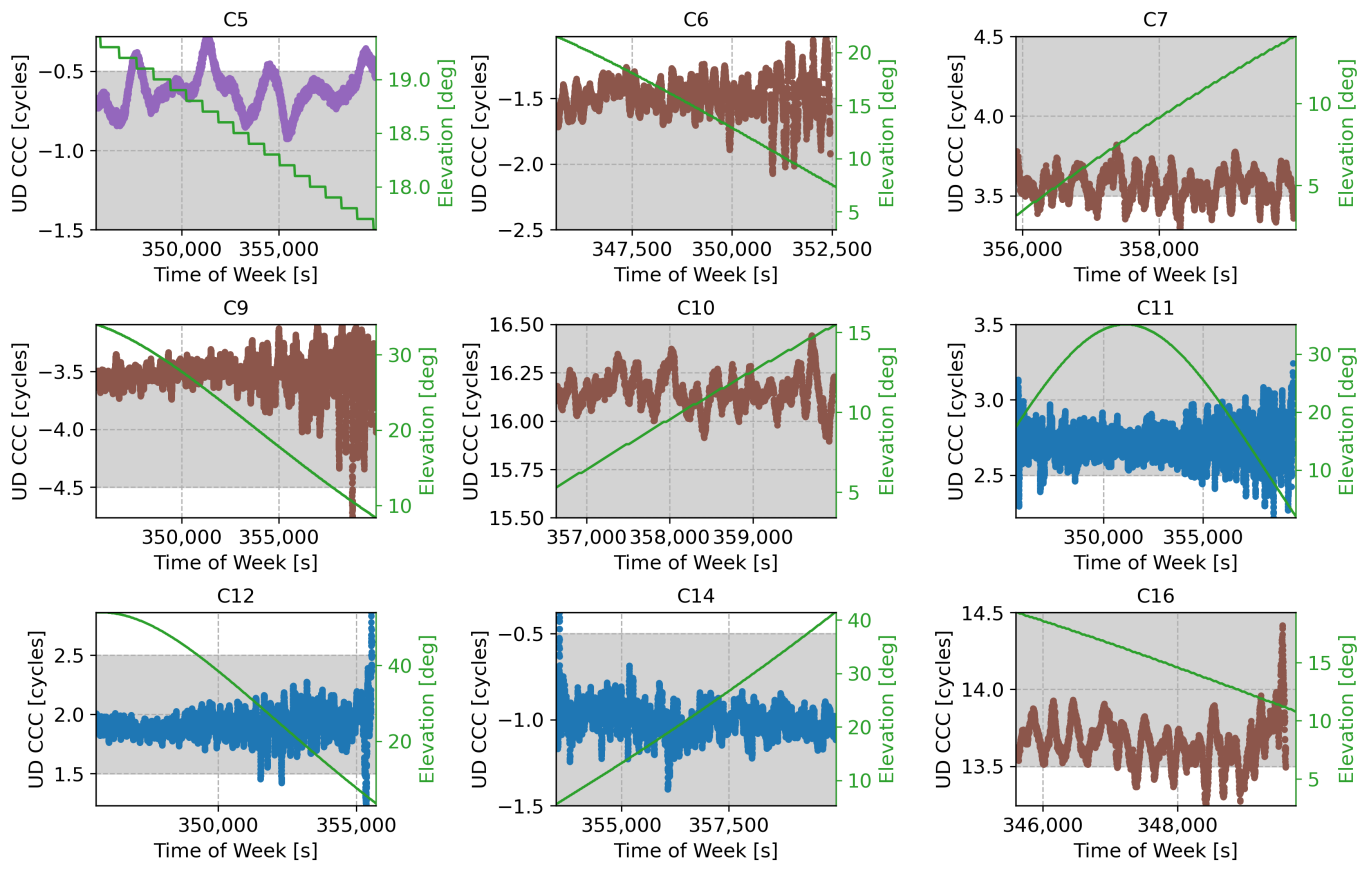
UD code-carrier combinations used in the ambiguity resolution process for the different satellites broadcasting both B2I and B3I signals, expressed in cycle counts. The corresponding satellite elevation is also provided in green. The satellite with a PRN of 5 is the GEO, whereas satellites 6, 7, 9, 10, and 16 are the IGSO satellites. The three remaining satellites are the MEO satellites.

The code-carrier combinations obtained for the B2I/B3I case are shown in Figure 15, where three types of satellites are analysed. The satellite with a PRN of 5 is the GEO, and its corresponding code-carrier combination is characterised by a significant bias, with a mean close to the half-cycle boundary. This made the ambiguity resolution process unreliable, as it allowed for the selection of two candidate integer values. The PRN 5 code-carrier combination was affected by smooth slow-varying oscillations, which were likely related to the dynamics of the GEO satellite. The results for the IGSO satellites are depicted in brown in Figure 15. In this case, significant biases were also observed, particularly for satellites with PRNs of 6, 7, 9, and 16. In such cases, the biases brought the code-carrier combinations close to the half-cycle boundaries, making the ambiguity resolution process unreliable. The code-carrier combinations of the satellite with a PRN of 10 exhibited a smaller bias, and the associated ambiguity could be reliably resolved.

The B2I/B3I code-carrier combinations obtained for the MEO satellites were affected by reduced biases, making them suitable for use in the meta-signal approach.

These results are confirmed by the summary statistics reported in Table 6. Among the MEO satellites, only the satellite with a PRN of 11 showed an FB significantly different from zero. Additional investigations are required to assess the consistency of the empirical results obtained.

### 5.4. The B3I/B1C Combination

Despite the previous results, it was not possible to obtain similar results with other combinations. Indeed, the remaining combinations are characterised by relatively short wavelengths, as reported in Figure 2. Given such short wavelengths and the presence of biases in the code-carrier combinations, it was not possible to reliably solve the ambiguity resolution process and effectively reconstruct the high-accuracy pseudoranges. In this section, the B3I/B1C combination is analysed. The results for the remaining combinations were very similar to the findings discussed here, and they are not reported in order to avoid unnecessary repetition.

**Figure 16:**
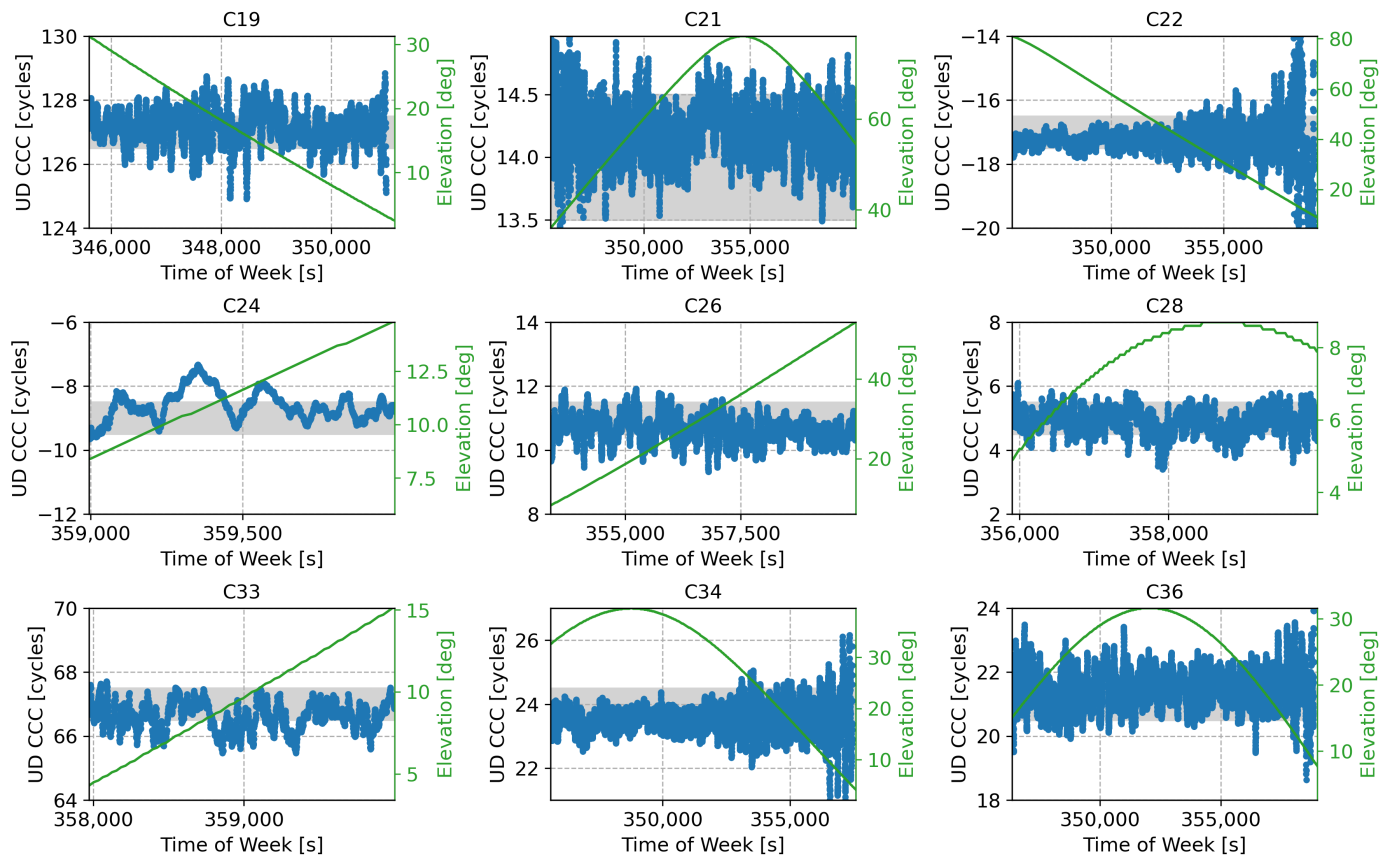
UD code-carrier combinations used in the ambiguity resolution process for the different satellites broadcasting both B3I and B1C signals, expressed in cycle counts. The corresponding satellite elevation is also provided in green.

The UD code-carrier combinations, expressed in cycle counts, are provided in Figure 16 for the different satellites broadcasting both B3I and B1C signals. In this case, the combinations were characterised by standard deviations of around 0.5 cycles and often exceeded the bands that would map the code-carrier combinations to a single integer value. Some of the combinations considered in Figure 16 were also affected by biases that further compromised the ambiguity resolution process.

This fact is confirmed by the summary values reported in Table 7. A common FB could not be identified. If a common FB were present, it could have been estimated and removed when forming the code-carrier combinations.

**Figure 17:**
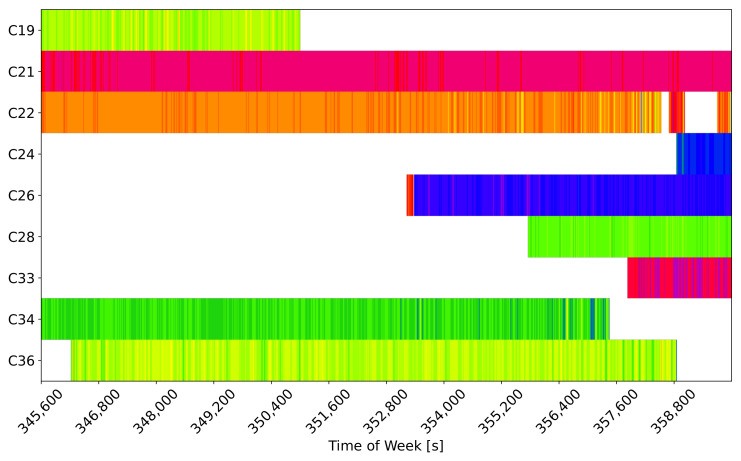
Graphical representation of the integer number of cycles estimated using the rounding operation in (11) for the different satellites. B3I/B1C case.

Under such conditions, it was not possible to lock on a single integer number of cycles and unambiguously reconstruct high-accuracy pseudoranges (11). This fact is confirmed in Figure 17, which provides a graphical representation of the integer number of cycles estimated for the B3I/B1C case.

**Figure 18:**
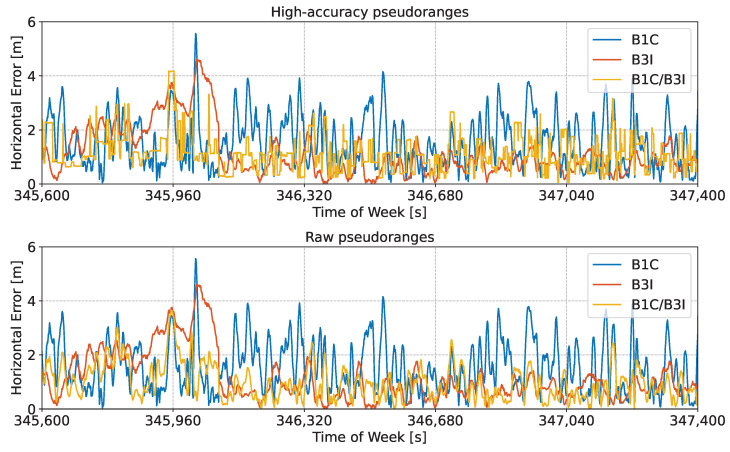
Time evolution of the horizontal error obtained using an SPP solution for different meta-signal measurements. The single side-band solutions are also provided for comparison purposes. B3I/B1C combination. (**Upper box**) Solution obtained using high-accuracy pseudoranges. (**Lower box**) Solution obtained using raw pseudoranges.

The estimated integer numbers of cycles often varied between two/three values for all the satellites considered in Figure 17. Thus, the corresponding high-accuracy pseudoranges would be affected by jumps and discontinuities in the order of the B3I/B1C wide-lane wavelength, which measured 0.98 m. In turn, these jumps and discontinuities would affect the final position solution, which is investigated in Figure 18.

The figure shows the time evolution of the horizontal error obtained using an SPP solution for the different meta-signal measurements obtained using the B3I and B1C signals. In this case, the high-accuracy pseudoranges did not lead to a significant improvement with respect to the best single side-band solution, which was obtained for the B3I signal. Jumps and discontinuities can be clearly seen in the horizontal errors of the navigation solution obtained using the high-accuracy pseudoranges and depicted in the upper part of Figure 18. Similar results were obtained for the vertical channel and are not repeated here.

The statistical parameters of the horizontal and vertical errors for the B3I/B1C configuration are summarised in Table 8. The results reported in the table confirm that in this case, the high-accuracy pseudoranges only provided a limited benefit. Although the raw pseudoranges provided some improvements with respect to the side-band solutions, the full benefits of the subcarrier could not be exploited in this case.

### 5.5. Other Combinations

The other Beidou meta-signal combinations introduced in Section 3 but not studied in the previous sections were characterised by wide-lane wavelengths around or lower than one metre. Under such conditions, it was difficult to reliably reconstruct the high-accuracy pseudoranges, and results similar to those discussed in Section 5.4 were observed. In order to avoid repetition of similar findings, only summary results are provided for these combinations.

**Figure 19:**
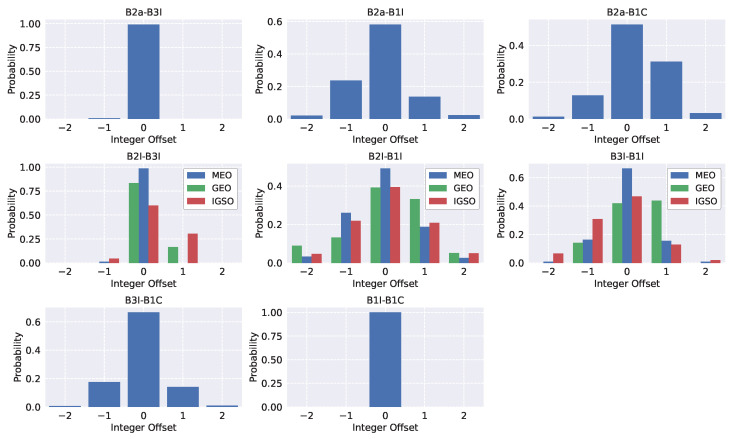
Probabilities of estimating different integer numbers of cycles using the rounding operation in (11). The x-axis, denoted as “Integer Offset”, refers to the offset with respect to the median value observed for the different satellites. Several meta-signal combinations are considered.

In particular, Figure 19 shows the probabilities of estimating different integer numbers of cycles for the different Beidou meta-signal combinations. The probabilities were estimated by using the measurements obtained from the test described in Section 4, considering all the satellites contributing to a specific code-carrier combination simultaneously. The zeroes on the x-axes in the different boxes in Figure 19 refer to the median integer number of cycles estimated for the different satellites.

More specifically, for each code-carrier combination, all available satellites were jointly considered. For each satellite, the median integer number of cycles estimated during the test was computed. Then, the probability that this median value, denoted as zero, was actually found through the rounding operation in (11) was determined. The probabilities of finding an integer value equal to the median plus the different offsets in the x-axes in Figure 19 were also estimated. The results estimated for each satellite were then averaged, leading to the histograms shown in Figure 19. Ideally, one would like to obtain a probability equal to one concentrated around zero. This would imply that the integer number of cycles can be resolved without ambiguity. This happened for the B1I/B1C case shown in the last row of Figure 19. Also, the B2a/B3I case led to high probabilities of ambiguously determining the associated integer number of cycles.

The combinations obtained for the BDS-2 satellites are considered in the second row of Figure 19. In this case, the results are categorised based on the satellite type. As highlighted in Section 5.3 for the B2I/B3I case, the code-carrier combinations obtained for the GEO and IGSO satellites were characterised by significant biases, which compromised the ambiguity resolution process and led to significant probabilities of observing integer offsets different from zero. For the MEO satellites and the B2I/B3I case, the ambiguity resolution process was mostly successful, and probabilities were concentrated around the zero offsets. Since only three MEO satellites were available for the analysis, these combinations, along with the study of the B2b/B3I case where the B2b signal replaced the B1I component, required additional investigations.

All the other combinations considered in Figure 19 were affected by significant probabilities of estimating an integer number of cycles different from the median value. Without appropriate mitigation techniques, these probabilities could lead to jumps and discontinuities in the final position solution, as in the B3I/B1C case discussed in Section 5.4. Under such conditions, the benefits of the meta-signal approach are limited.

## 6. Discussion

BDS, with its second- and third-generation signals, provides a wide range of dual-frequency combinations with different frequency separations, ranging from a few MHz to several hundred MHz. Eight such combinations were analysed in this paper. From the analysis, it emerged that unambiguous meta-signal measurement reconstruction could be achieved using several BDS signal combinations with frequency separations up to 90 MHz.

The B1I/B1C combination was characterised by a wavelength of more than twenty metres. In these cases, ambiguities could be reliably solved, leading to unambiguous high-accuracy pseudoranges. The reconstructed B1I/B1C measurements allowed for improved positioning performance, reducing both horizontal and vertical errors compared to the single side-band solutions. Experiments conducted on the B2a and B3I signals showed that in this case, ambiguities could also be reliably solved, leading to very accurate pseudorange measurements. This combination was characterised by a frequency separation of about 90 MHz. Thus, the reconstructed measurements were characterised by a very wide Gabor bandwidth, which, in turn, led to very accurate pseudorange measurements. This was clearly visible in the position solution obtained using the B2a/B3I reconstructed observations. The B2I/B3I combination was also analysed. Preliminary tests showed that this combination is a candidate for the successful implementation of the meta-signal reconstruction approach, as shown in the case of the MEO satellites. The measurements from the IGSO and GEO satellites were affected by biases that compromised the reconstruction process.

The analysis of the ACE-BOC combination was not possible since the receiver used for the test did not support the B2b component. However, its side-band components are separated by only 30.69 MHz and should lead to a reliable meta-signal reconstruction. Also, the B2b/B3I combinations, which were characterised by a frequency separation of about 60 MHz, are of interest. The analysis of the ACE-BOC and the other combinations involving the B2b signal is left for future work.

The other Beidou meta-signal combinations were characterised by larger frequency separations and, in such cases, it was not possible to reliably reconstruct the meta-signal pseudoranges. The ambiguity problem is common in several meta-signal processing approaches that are limited by the frequency separation of the side-band components. Future work will investigate the potential of smoothing and cycle slip detection for improving the ambiguity resolution process in meta-signal measurement reconstruction. The impact of higher user dynamics and a more significant ionospheric activity will also be considered.

## 7. Conclusions

In this paper, the synthetic meta-signal reconstruction approach has been applied to Beidou second- and third-generation signals. In this approach, wideband meta-signal observations are reconstructed using measurements from two different frequencies, denoted as side-band components. The final high-accuracy meta-signal pseudoranges are reconstructed by combining code and carrier measurements. Although the adoption of the carrier information has the potential to significantly improve the quality of the final pseudoranges, it also entails an ambiguity resolution process. The effectiveness of the meta-signal approach depends on the success of the ambiguity resolution process, which is implemented through a rounding operation. Factors influencing the ambiguity resolution process are the frequency separation between side-band components and the presence of differential biases in the side-band measurements. 

## Figures and Tables

**Table 1:** Different Beidou meta-signal combinations and the associated satellite visibility conditions.

Combination	Satellites	Combination	Satellites
B2a/B3I	BDS-3	B2I/B3I	BDS-2
B2a/B1I	BDS-3	B2I/B1I	BDS-2
B3I/B1I	BDS-2 and 3	B2a/B1C	BDS-3
B3I/B1C	BDS-2 and 3	B1I/B1C	BDS-3

**Table 2:** Summary statistics for the B1I/B1C UD code-carrier combinations obtained for the different satellite signals considered in the test, expressed in cycle counts.

Satellite	CCC Average	CCC FB	CCC Std
19	4.310	0.31	0.039
21	8.297	0.297	0.012
22	11.299	0.299	0.036
24	61.300	0.300	0.026
26	−23.704	0.296	0.025
28	20.296	0.296	0.024
33	−3.692	0.308	0.029
34	−3.697	0.303	0.027
36	−25.687	0.313	0.033
Average		0.302	0.028

**Table 3:** Summary statistics for the positioning errors obtained considering the B1I/B1C combination. “Raw” denotes the solution obtained from raw meta-signal pseudoranges, and “HA” denotes the high-accuracy solution.

	Mean Absolute Error (m)	RMS Error (m)	Standard Deviation (m)
Horizontal Component			
B1I	1.329	1.565	0.826
B1C	1.617	1.876	0.951
B1I/B1C Raw	1.023	1.215	0.655
B1I/B1C HA	0.874	1.061	0.602
B1I/B1C HA with pre-correction	0.888	1.062	0.583
Vertical Component			
B1I	1.008	1.306	0.832
B1C	1.267	1.562	0.913
B1I/B1C Raw	0.774	1.022	0.668
B1I/B1C HA	0.732	0.955	0.615
B1I/B1C HA with pre-correction	0.749	0.993	0.652

**Table 4:** Summary statistics for the B2a/B3I UD code-carrier combinations obtained for the different satellite signals considered in the test, expressed in cycle counts.

Satellite	CCC Average	CCC FB	CCC Std
19	−10.087	−0.087	0.191
21	9.061	0.061	0.056
22	42.033	0.033	0.121
24	−4.968	0.032	0.115
26	−3.192	−0.192	0.129
28	−3.87	0.13	0.090
33	53.1	0.1	0.089
34	3.009	0.009	0.138
36	10.898	−0.102	0.187
Average		−0.002	0.124

**Table 5:** Summary statistics for the positioning errors obtained considering the B2a/B3I combination. “Raw” denotes the solution obtained from raw meta-signal pseudoranges, and “HA” denotes the high-accuracy solution.

	Mean Absolute Error (m)	RMS Error (m)	Standard Deviation (m)
Horizontal Component			
B2a	1.793	1.999	0.882
B3I	1.148	1.469	0.916
B2a/B3I Raw	0.717	0.862	0.478
B2a/B3I HA	0.644	0.743	0.370
Vertical Component			
B2a	0.888	1.090	0.632
B3I	0.978	1.193	0.682
B2a/B3I Raw	0.641	0.774	0.434
B2a/B3I HA	0.434	0.443	0.112

**Table 6:** Summary statistics for the B2I/B3I UD code-carrier combinations obtained for the different satellite signals considered in the test, expressed in cycle counts.

Satellite	Type	CCC Average	CCC FB	CCC Std
5	GEO	−0.62	0.38	0.12
6	IGSO	−1.48	−0.48	0.14
7	IGSO	3.562	−0.438	0.091
9	IGSO	−3.517	0.483	0.17
10	IGSO	16.155	0.155	0.085
11	MEO	2.712	−0.288	0.103
12	MEO	1.909	−0.091	0.118
14	MEO	−1.002	−0.002	0.09
16	IGSO	13.657	−0.343	0.144
Average			−0.069	0.118

**Table 7:** Summary statistics for the B3I/B1C UD code-carrier combinations obtained for the different satellite signals considered in the test, expressed in cycle counts.

Satellite	CCC Average	CCC FB	CCC Std
19	127.167	0.167	0.565
21	14.207	0.207	0.213
22	−17.265	−0.265	0.666
24	−8.595	0.405	0.470
26	10.662	−0.338	0.418
28	4.887	−0.113	0.402
33	66.619	−0.381	0.451
34	23.534	−0.466	0.479
36	21.504	−0.497	0.602
Average		−0.142	0.474

**Table 8:** Summary statistics for the positioning errors obtained considering the B3I/B1C combination. “Raw” denotes the solution obtained from raw meta-signal pseudoranges, and “HA” denotes the high-accuracy solution.

	Mean Absolute Error (m)	RMS Error (m)	Standard Deviation (m)
Horizontal Component			
B1C	1.617	1.876	0.951
B3I	1.148	1.469	0.916
B3I/B1C Raw	0.978	1.169	0.640
B3I/B1C HA	1.128	1.311	0.667
Vertical Component			
B1C	1.267	1.562	0.913
B3I	0.978	1.193	0.682
B3I/B1C Raw	0.760	0.920	0.462
B3I/B1C HA	0.880	1.098	0.694

## Data Availability

The data presented in this study are available on request from the corresponding author.
